# Nitrous Oxide Effect on Relieving Anxiety and Pain in Parturients Under Spinal Anesthesia for Caesarean Section

**DOI:** 10.5812/aapm.16662

**Published:** 2014-05-26

**Authors:** Nahid Manouchehrian, Mohammad Hossein Bakhshaei

**Affiliations:** 1 Department of Anesthesiology, Fatemieh Hospital, Hamadan University of Medical Sciences, Hamadan, Iran; 2 Department of Anesthesiology, Ekbatan Hospital, Hamadan University of Medical Sciences, Hamadan, Iran

**Keywords:** Spinal Anesthesia, Anxiety, Cesarean Section, Nitrous Oxide, Visual Analogue Scale

## Abstract

**Background:**

Anxiety is an unpleasant experience that may have adverse effects on the process of anesthesia, cesarean delivery, recovery period and postoperative pain. Anxiety can also affect maternal satisfaction of the medical cares that provided by the medical team.

**Objectives:**

To compare the effects of inhalational 50% nitrous oxide (N_2_O) with oxygen on reducing anxiety and pain in parturients who have undergone caesarean section under spinal anesthesia.

**Patients and Methods:**

In this double-blind clinical trial, 56 primigravid parturients were randomly assigned into two groups according to the operating list schedule. The experimental group received inhalational 50% N_2_O three minutes before spinal anesthesia to the end of delivery. The control group received only oxygen. Flow meters were covered by a dark shield and monitored by an experienced nurse anesthetist. Pain and anxiety of patients were measured using visual analogue scale (VAS) by another nurse who was neither involved in the anesthetic process nor aware of the participants' allocation and inhalation agents. Data regarding sedation level, ephedrine use, nausea, vomiting, and neonate Apgar score were recorded as well.

**Results:**

Overall mean ± SD of anxiety VAS scores was 1.77 ± 1.5 in the experimental group and 3.12 ± 1.73 in the control groups (P = 0.003). The mean ± SD of pain VAS scores of the experimental and control groups were 0.82 ± 1.5 and 1.64 ± 1.45, respectively (P = 0.042). No significant differences were seen regarding blood oxygen saturation, neonate Apgar scores, total used ephedrine, operation time, delivery time, nausea, and vomiting between the two studied groups.

**Conclusions:**

Inhalation of 50% N_2_O can significantly decrease anxiety (without clinically significant side effects) compared with O_2_ inhalation in parturients who have undergone caesarean section under spinal anesthesia.

## 1. Background

Caesarean section is a relatively common procedure to terminate pregnancy. Currently, it is performed under spinal anesthesia, which has fewer complications compared with the general anesthesia. Anxiety is an unpleasant experience that may have adverse effects on the process of anesthesia, caesarean delivery, recovery, and postoperative pain. Anxiety can also affect maternal satisfaction with regard to the medical care (1).

 It has been reported that performing caesarean section under spinal anesthesia is associated with too much anxiety (2, 3). This very fact that caesarean section is performed on the patient's abdomen while she is awake can cause anxiety (3, 4). Therefore, relieving anxiety may change caesarean section and childbirth into a pleasant experience (1).

One of the medications which its anxiolytic effects have been studied in dental procedures and on patients with cancer is nitrous oxide (N_2_O) (3-5). Administration of 50% N2O causes analgesia and reduces fear of pain and anxiety in patients, but has mild and self-limited side effects (6-8). Considering the adverse effects of anxiety on patients and also the side effects of usual anxiolytic medications, efforts to achieve an effective modality to treat anxiety in pregnant women who undergo caesarean section are important.

## 2. Objectives

We aimed to assess the anxiolytic effects of inhalational 50% N_2_O in primigravid parturients who were candidates for elective caesarean section under spinal anesthesia.

## 3. Patients and Methods

### 3.1. Study Population

This study was a double-blind, randomized, clinical trial performed at a teaching University hospital (affiliated to Hamadan University of Medical Sciences, Hamadan, Iran) during 2008-2009. The protocol of the study was approved by the Ethics Committee of the University. The participants consisted of 56 primigravid parturients who were candidates for elective caesarean section under spinal anesthesia and had T4–T6 spinal anesthesia level. Exclusion criteria were as follows: personality disorder, addiction, complicated pregnancy, including preeclampsia, diabetes mellitus, hypothyroidism and hyperthyroidism, chronic obstructive pulmonary disease, valvular heart disease, upper respiratory tract infection or sinus obstruction, recent history of middle or inner ear disease or surgery, and history of asthma.

**Figure 1.**
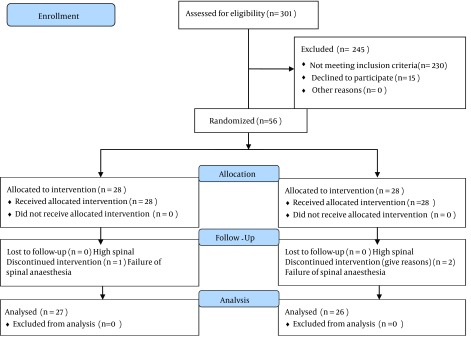
The Flowchart of the Trial

 The participants were assigned into two groups according to the block randomization method. After obtaining the written informed consent, participants in the N_2_O group (28 parturients) received 50% N_2_O during spinal anesthesia and caesarean section, and the control group (28 parturients) received just oxygen. Flowmeters were covered by a dark shield, and an experienced anesthesia nurse monitored them continuously. Figure 1 shows the flowchart of the trial.

### 3.2. Anxiety and Pain Assessment

For the measurement of anxiety, we used a visual analogue scale (VAS), designed as a 10-cm horizontal line (scored from 0 to 10). After explaining the procedure for the patients, they were asked to mark their anxiety on the scale (0 = no anxiety to 10 = maximum anxiety). A similar VAS was used for pain measurment. Anxiety and pain assessments were performed in six stages:

1. before entering the operating room,
2. after entering the operating room and placing on the operating table,
3. during induction of spinal anesthesia,
4. during skin and uterus incision,
5. at the time of delivery, and
6. at the end of the caesarean section and upon transfer of the patient to the recovery unit.

Maternal blood pressure and heart rate were measured before and after inducing the spinal anesthesia and then every 5 minutes up to 15 minutes and every 15 minutes up to the end of the operation. Pain and anxiety of the patients were measured by a nurse who was uninvolved in the anesthesia process and unaware of the participants' allocation and inhalational agents.

### 3.3. Anesthesia Method

All parturients after receiving 10 mL/kg serum Ringer underwent spinal anesthesia in sitting position using 25-gauge Quincke spinal needle by median approach and then 2.5 ml of bupivacaine 0.5% (12.5 mg) was injected intrathecally. The sensory block levels were assessed by pinprick sensation before surgery the patients with sensory block levels T4-T6 were included in our study. All steps of anesthesia were performed by a single anesthesiologist who was not aware of the study protocol.

Three minutes before inducing spinal anesthesia, 50% N_2_O (3 liters of nitrous oxide and 3 liters of oxygen by anesthesia mask) was administered to the N_2_O group and O_2_ (6 liters of oxygen by anesthesia mask) for the control group by a semi-closed anesthesia circle system. Nitrous oxide or oxygen administration was continued until delivery.

In order to assess the patient’s sedation, we used the Ramsay Sedation Scale (RSS) (9). This scale is used for evaluating the sedation rate in hospital inpatients and categorized in six scores as follows:

1. anxious, restless, agitated;
2. cooperative and calm;
3. responsive to commands only;
4. brisk response to stimulus;
5. sluggish response to stimulus; and
6. unresponsive.

For hemodynamic assessment, maternal vital signs, arterial oxygen saturation percentage (SPaO_2_, using pulse oxymetry A520, Oxypleth, Germany), nausea, vomiting, amnesia, total dosages of ephedrine used, any indication of caesarean section, level of spinal anesthesia, duration of surgery, delivery duration, as well as demographic data were recorded. The neonates Apgar score were recorded as well. The Apgar scoring system (which is based on physiological responses of a neonate to birth), is a good method for determining the need of a neonate for resuscitation. We measured and documented this score 1 and 5 minutes after delivery (6, 7).

 Sample size was determined at 95% confidence interval and a difference in the expected mean anxiety scores in cases and controls (μ 1 - μ 2 = 14) with standard deviation of 11 (taken from previous study of Valleio et al. (3)) which estimated to be at least 28 patients.

The results were reported as mean ± standard deviation (SD) for the quantitative variables and percentages for the categorical variables. The groups were compared using the Student t test for the continuous variables and the chi-square test (or the Fisher's exact test if required) for the categorical variables. Correlation between the variables was examined by the Pearson’s correlation coefficient test. A P value ≤ 0.05 was considered statistically significant. All the statistical analyses were performed using SPSS version 15.0 (SPSS Inc., Chicago, IL, USA) for Windows.

## 4. Results

Average age of the parturients was 23.15 ± 4.01 years. Mean weight of neonates at the time of birth was 3.079 ± 0.304 kg. Table 1 presents the characteristics of the two studied groups. The mean ±SD of anxiety VAS scores in the N2O and control groups were 6.22 ± 3.29 and 5.21 ± 3.20, respectively before induction of spinal anesthesia with no significant discrepancy. Overall mean ± SD of anxiety VAS scores (including all six stages studied) in N_2_O and control groups were 1.77 ± 1.5 and 3.12 ± 1.73, respectively that was significantly higher in the latter group (P = 0.003).

**Table 1.** Comparison of Clinical Characteristics of the Two Studied Groups^a^

-	Experimental Group, Nitrous Oxide	Control group, Oxygen	P Value
**Age, y**	25.37 ± 4.51	24.89 ± 3.51	NS
**Level of anesthesia**	-	-	NS
T3	4	9	-
T4	19	17	-
T5	1	0	-
T6	3	0	-
**Child birth weight, gram**	3153 ± 295	2995 ± 295	NS
**SaO**_**2**_	97.94 ± 0.89	97.94 ± 0.74	NS
**Ephedrine dosage used, mg**	14.82 ± 13.83	11.85 ± 11.77	NS
**Cesarean duration, min**	41.75 ± 10	41.67 ± 9.5	NS
**Child delivery duration, min**	3.9 ± 1.5	5.6 ± 2	0.003
**Neonates apgar, 1** **minute**	8.14	8.63	0.239
**Neonates apgar, 5** **minutes**	9.36	9.69	0.701

^a^ Abbreviations: SaO_2_, arterial blood oxygen saturation; NS, not significant.

**Figure 2.**
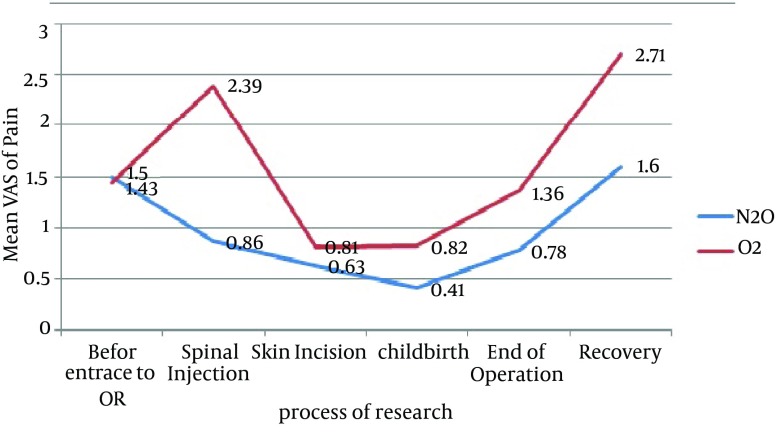
Mean Visual Analogue Scale of Pain According to Six Stage of Research Process

The highest mean of anxiety VAS score in N_2_O group was registered at the time of spinal injection (5.54) and the lowest was at the time of recovery (0.44). Similarly; in the control group, the highest anxiety VAS score was recorded at the time of spinal injection (5.57), and the lowest was at the time of recovery (0.50). As shown in Table 2, in three stages, including skin incision, childbirth, and at the end of surgery, anxiety was significantly lower in the N_2_O group compared with the control group. The mean ± SD of pain VAS scores before inducing spinal anesthesia were 1.5 ± 2.43 in the N_2_O and 1.43 ± 2.45 in the control group with no significant difference (P = 0.912). Total mean ± SD of pain VAS scores (including all six stages studied) were 0.82 ± 1.5 in N2O group and 1.64 ± 1.45 in control group (P = 0.042) (Figure 2).

**Table 2.** Comparison of Mean Anxiety Visual Analogue Scale Scores Between the Two Groups in Different Anesthesia Stages

	Experimental Group , Nitrous Oxide	Control Group ,Oxygen	P Value
**Spinal injection**	5.54	5.57	0.211
**Skin incision**	2.50	5.18	0.001
**Childbirth**	0.59	2.93	0.001
**Operation termination**	0.41	1.43	0.028
**Recovery**	0.44	0.50	0.840

As shown in the figure, the highest mean pain VAS score in the N_2_O group was at the time of recovery (1.6) and the lowest was at the time of childbirth (0.41). In the control group, the highest mean of pain VAS score was at the time of the recovery (2.71), and the lowest was at the time of skin incision (0.81). Only in the stage of spinal injection, the mean pain VAS score was lower in the N_2_O group compared with the control group (P = 0.013).

The mean ± SD level of sedation was 2 ± 0.25 in the N_2_O group and 1 ± 0.27 in the control group that was higher in the former (P < 0.001). In the N_2_O group, the sedation level was a little more than the control group; however, it was not beyond scale 2. About 55.6% of the parturients in the N_2_O group and 39.3% of the controls had intraoperative nausea with no significant difference. Only one patient had nausea after the surgery. No significant difference was found between the two studied groups with respect to the maternal systolic blood pressure, maternal heart rate, dose of ephedrine, and Apgar score of the neonates (minutes 1 and 5).

## 5. Discussion

We found beneficial effects for inducing nitrous oxide over oxygen in terms of reducing childbirth anxiety and pain in primigravid women who underwent caesarean section under spinal anesthesia. In all stages of anesthesia, VAS scores showed that anxiety was more severe in the control group compared with the experimental group, particularly during skin incision, childbirth, and at the end of operation. Although there are reports regarding the beneficial effects of N_2_O on pain, fear, and anxiety of the patients undergoing dental procedures or among patients with cancer, there are few studies about such beneficial effects of N_2_O during caesarean section (3-5).

Vallejo and colleagues studied the effects of N_2_O on anxiety and pain of 30 patients who were candidate for caesarean section (3). They reported that the mean VAS anxiety score measured in the N_2_O group was significantly lower than the O_2_ group, which is consistent with our results. Furthermore, they examined the difference between anxious (anxiety VAS > 50) and calm participants (anxiety VAS < 50); they concluded that in the first group, N_2_O administration significantly decreased anxiety VAS, but in low anxiety group no significant difference was observed between N_2_O and O_2_ groups.

 Vallejo and colleagues also compared the rate of VAS pain in the two groups, but found no significant difference between the two groups in mean pain scores (3). Their findings were different from ours because we found that mean pain VAS scores (including all six stages studied) in the experimental group were lower than the control group.

Kangasundarm and associates (10) aimed to assess the effect of 50-70% N_2_O on decreasing pain and anxiety in 90 children who were frequently undergoing painful processes such as bone marrow biopsy or dressing change. They used the Observational Scale of Behavioral Distress-Revised (OSBD-R) to evaluate anxiety. They reported that children who aged more than 6 years, experienced less anxiety after using N_2_O.

A study evaluated the effect of N_2_O on anxiety-like behavior and pain among rats. They concluded that administration of N_2_O would decreas anxiety-like behavior in rats (11).

Beneficial effects of N2O administration have been reported with respect to alleviating pain too. Former studies have shown such effects, mainly among pediatric patients. Nitrous oxide has been effective as an intramuscular analgesic for the treatment of fractures with a more rapid onset and higher patient satisfaction (12, 13). The analgesic effect of N_2_O during labor is known for a long time, and it is used widely in different countries (14). Maximum analgesic effect was reported in 70% concentration (15).

Our results regarding the sedation level showed that N_2_O has induced increased drowsiness in pregnant women, but this drowsiness was not clinically significant. Nitrous oxide did not have significant effects on the caesarean process and operating duration. Delivery duration was also similar between the two groups. Even the delivery time was shorter in N_2_O received parturients. No significant complication was seen in the study and only one patient in the N_2_O group experienced severe nausea and vomiting, which were resolved with hydration.

There are concerns about possible effects of N_2_O on arterial blood oxygen desaturation or increasing emesis in recipients. We did not find any oxygen desaturation in the studied parturients. This finding has also been noted in a randomized clinical trial which showed self-administered 50% N2O did not seem to predispose parturients to hemoglobin oxygen desaturation (16).

Anxiety is an unpleasant emotion and may affect the operation process; also anxiety is normally concomitant with pain. There are many methods for detecting anxiety before operation, but we used VAS for determination of anxiety before operation like Kindler et al. and Dubois D et al. did (17-19). Administration of N_2_O can significantly decrease anxiety and pain in parturients who inhale 50% concentration of this agent by facemask in comparison with those who received oxygen in the control group. No clinically important side effect such as hypotension, severe nausea and vomiting, or arterial blood oxygen desaturation was seen in N_2_O-received parturients.
